# Comparison of the C-Reactive Protein Level and Visual Analog Scale Scores between Piezosurgery and Rotatory Osteotomy in Mandibular Impacted Third Molar Extraction

**DOI:** 10.3390/life12060923

**Published:** 2022-06-20

**Authors:** Lakshmi Shetty, Khushal Gangwani, Uday Londhe, Swati Bharadwaj, Mohammed Mousa H. Bakri, Ahmed Alamoudi, Rodolfo Reda, Shilpa Bhandi, A. Thirumal Raj, Shankargouda Patil, Luca Testarelli

**Affiliations:** 1 Department of Oral and Maxillofacial Surgery, Dr. D. Y. Patil Dental College and Hospital, Dr. D. Y. Patil Vidyapeeth, Pune 411018, India; lacchu33@yahoo.co.in (L.S.); khushalhkhr@gmail.com (K.G.); uday.londhe@dpu.edu.in (U.L.); swatirbharadwaj@gmail.com (S.B.); 2 Department of Oral and Maxillofacial Surgery and Diagnostic Sciences, College of Dentistry, Jazan University, Jazan 45142, Saudi Arabia; mmb644@nyu.edu; 3 Department of Oral Biology, Faculty of Dentistry, King Abdulaziz University, Jeddah 21589, Saudi Arabia; ahmalamoudi@kau.edu.sa; 4 Department of Oral and Maxillo Facial Sciences, Sapienza University of Rome, 00161 Rome, Italy; luca.testarelli@uniroma1.it; 5 Department of Restorative Dental Science, Division of Operative Dentistry, College of Dentistry, Jazan University, Jazan 45142, Saudi Arabia; shilpa.bhandi@gmail.com; 6 Department of Cariology, Saveetha Dental College & Hospitals, Saveetha Institute of Medical and Technical Sciences, Saveetha University, Chennai 600077, India; 7 Department of Oral Pathology and Microbiology, Sri Venkateswara Dental College and Hospital, Chennai 600130, India; thirumalraj666@gmail.com; 8 Department of Maxillofacial Surgery and Diagnostic Sciences, Division of Oral Pathology, College of Dentistry, Jazan University, Jazan 45142, Saudi Arabia; 9 Centre of Molecular Medicine and Diagnostics (COMManD), Saveetha Dental College & Hospitals, Saveetha Institute of Medical and Technical Sciences, Saveetha University, Chennai 600077, India

**Keywords:** c-reactive protein, third molar extraction, pain, piezosurgery, rotatory burs

## Abstract

This study aimed to compare the C-reactive protein level and visual analog scale scores of piezo- and rotatory-based surgical extraction of the third molar. As a split-mouth study, the comparative groups consisted of 25 patients, each of whom underwent surgical removal of the third molar by piezo on one side and rotatory bur on the other side. C-reactive protein levels were quantitatively assessed (enzyme-linked immunosorbent assay) before and immediately post-extraction. The immediate postoperative blood sample (baseline) C-reactive protein levels were compared with 24 h and 72 h post-op samples, both within and between the groups. Pain was assessed using the visual analog scale at 24 h and 72 h post-operatively. The C-reactive protein levels were lower in the piezo group than in the rotatory group, although the difference was not significant (*p* > 0.05). The visual analog scale score was significantly (*p* < 0.01) lower in the piezo group than in the rotatory group. The C-reactive protein levels increased in both the rotary and piezo groups from the pre-op to the immediate post-op value, but in the piezo group, the levels dropped back after 24 h. On the contrary, in the rotatory group, the C-reactive level kept increasing until 24 h; the visual analog scale score dropped significantly from 24 to 72 h for both the rotatory and piezo groups. Surgical techniques that could spare the surrounding soft tissues, such as the piezo, could aid in reducing overall postoperative morbidity.

## 1. Introduction

Third molar extraction is one of the most common surgeries in any dental practice. The most common postoperative sequelae include pain and swelling [1]. The primary reason for these signs and symptoms is the acute inflammation that occurs due to the surgical handling of soft and hard tissues [2]. Such postoperative symptoms can be predicted by several factors, including peri-operative infection, pericoronitis, the difficulty of impaction, duration of the surgery, the technique of removal, perioperative use of antibiotics, etc. [3,4]. Given these wide ranges of variables, from the surgical technique employed to the optimal use of antibiotics, researchers have used specific markers of postoperative morbidity to compare and identify a better methodology. C-reactive protein (CRP) is one such marker. CRP is stimulated due to surgery-induced inflammation or any associated trauma/infection. The physiological value of CRP is <10 mg/L in healthy individuals. This value increased by double after 8 h of surgery and returned to the baseline value in a week [5,6]. CRP quantification is often used as a biomarker of systemic inflammatory status [7,8,9].

Osteotomy, the technique used for the 3rd molar removal, is technique-sensitive, especially in the field of maxillo-facial surgery. It involves drilling or cutting by osteotomes, which are relatively rough instruments, especially those that are rotatory based. Due to the exceedingly high temperatures produced during osseous drilling, osteotomies could result in marginal osteonecrosis and hamper bone regeneration. Piezosurgery, or piezoelectric bone surgery, is a relatively precise system for hard tissue removal. The piezo-associated ultrasound frequency does not damage the associated soft tissues. Catuna et al. (1953) published their work regarding the effects of ultrasonic waves on cutting hard tissues of dental origin [10]. Since then, the use of ultrasonics in dentistry, especially in periodontology and endodontics, has risen exponentially. The application of ultrasound methods in oral surgery was first introduced by Horton et al. at the end of the 1970s [11]. Due to the piezoelectric effect of ultrasonic bone-cutting instruments, micro-vibrations are produced in the bone. This fact was first established and described in 1880 by Marie and Jean Curie. A more advanced device working on the principle of ultrasonic vibrations was established in 1988 by Tomaso Vercellotti, an Italian maxillofacial surgeon, to overcome the limitations of conventional instrumentation in oral and maxillofacial hard tissue surgery [12]. This technique is not only clinically effective but also shows a more favorable tissue response in terms of the histological indications of wound healing and osteogenesis than other conventional bone removal techniques [13]. In addition, the shock waves produced in the fluid medium aid in diminishing the bacterial load at the site of operation [14]. The literature has been published on the use of piezo technology in dentistry for maxillary sinus lift procedures [15], bone harvesting [16], alveolar crest expansion, implantology [17], periodontal surgery, orthognathic, maxillofacial surgery [18], and dental extractions [19]. 

Given the increasing demand to employ piezo-surgery, the present study was undertaken to assess if the postoperative morbidity associated with 3rd molar surgery was lower in piezo-surgery compared to the rotatory bur technique. The CRP values in the blood and the visual analog scale for pain were assessed between the two surgical techniques at varying time points. The C-reactive protein was used for quantitative analysis to assess the changes for the first time and to determine the effects of the two different techniques for the removal of bone.

## 2. Materials and Methods

This original prospective study was conducted on patients requiring bilateral third molar extraction between April 2015 and April 2018. Written informed consent was obtained from the patients for the study. Dr. D. Y. Patil Vidyapeeth’s institutional ethics committee approval (DPU/Research/159(7)/2013, research proposal no—DR-1—2012–2013) was obtained on 30-03-2013.

Inclusion criteria: Healthy patients in the age range of 20–30 years requiring surgical removal of a bilateral mesioangular impacted lower third molar of the same difficulty index were selected. As a pre-operative elevated CRP value would interfere with the study results, only patients with a negative baseline (pre-operative) CRP qualitative test [RHELAX CRP; Tulip Diagnostics] were included in the study. For the CRP qualitative assessment, 2 mL of the patient’s venous blood was collected before the 3rd molar extraction. An elevated CRP concentration (above 6 mg/L) leads to visible agglutination, demonstrating a positive test. 

Exclusion criteria: Patients with any history of neurological disturbance and trauma, history of surgical procedures in the area of the third molar, pre-existing inflammation, or recent history of anti-inflammatory drugs or steroids intake, preoperatively (baseline) positive CRP qualitative test. 

Group A—25 sites surgical removal by Piezosurgery

Group B—25 sites surgical removal with rotatory osteotomy.

The split-mouth study design was adapted 25 patients were divided into 2 groups.

Group A = 25 (one side) mesioangular impacted mandibular teeth removed by piezosurgery

Group B = 25 (mesioangular impacted mandibular tooth removed by Rotatory osteotomy

No. of sides total n = 50

**Figure 1:**
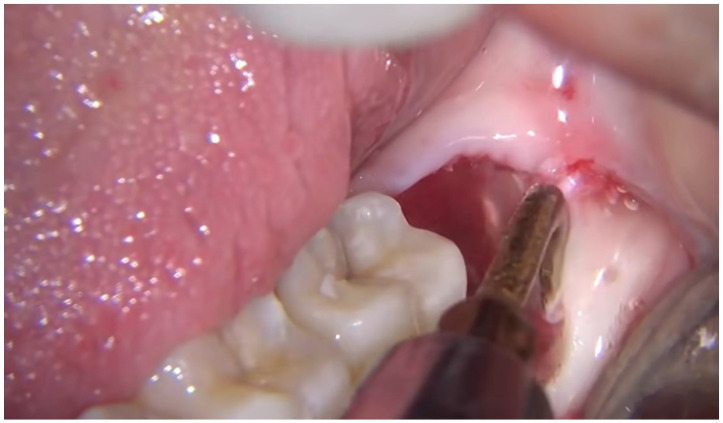
Surgical removal of the impacted tooth with piezosurgery.

The patients were allocated by simple random sampling using the SNOSE technique. Surgical removal with piezosurgery or rotary was randomly allocated with double-blinding of both the investigator and evaluator. The period between the surgeries in the same patient was 21 days. Their case history was recorded pre-operatively, and the blood was withdrawn for CRP level (baseline) and assessed using enzyme-linked immunosorbent assay (ELISA) [Spin React, Tulip Diagnostics]. These pre-operative quantitative values were considered baseline values. The 25 patients underwent surgical removal of the third molar by rotatory bur on one side and piezo on the other side, with a gap of 21 days between the surgeries. Using local anesthesia, the third molar (Figure 1) was surgically removed using the above-mentioned bone-cutting methods. Piezosurgery was conducted with a DMETEC surgystar with EXO1, EXO2, and EXO3. The rotatory osteotomy technique was done using a No. 10 bur and 703 SS white bur. Immediately post-surgery, a second blood sample was obtained. The CRP levels of these samples were estimated by ELISA. The third and fourth blood samples were collected at 24 and 72 h postoperatively using ELISA for the quantitative analysis of CRP. The Visual Analog Scale (VAS), which was a subjective evaluation, was used to assess postoperative pain at 24 h and 72 h. The patients were given the same anti-inflammatory drug (ibuprofen 400 mg) in both procedures for 3 days (once every 8 h) after the surgical procedure. The patients were followed for postoperative follow-up to check for postoperative inflammation in both groups. The statistical analysis was done with a paired *t*-test and ANOVA.

Statistical analysis: The obtained data were compiled on an MS Office Excel Sheet (v 2010, Microsoft Office, Washington, DC, USA) and subjected to statistical analysis using a Statistical Package for the Social Sciences (SPSS v 21.0, IBM, Chicago, IL, USA). Intragroup comparison of VAS across various time intervals was assessed using a paired *t*-test, while CRP was assessed using repeated-measures ANOVA. An intergroup comparison of VAS and CRP between both groups was done using a *t*-test. For all the statistical tests, *p* < 0.05 was considered statistically significant, keeping α error at 5% and β error at 20%, thus designating 80% power to the study.

## 3. Results

The average durations of the piezo and rotary bur techniques were 50 min and 25 min, respectively.

Intra-group comparison: CRP value within the piezo group showed a difference from immediate post-op to 24 h, and 72 h post-op, although the difference was not statistically significant (*p* > 0.05). The CRP value within the rotatory group showed a statistically significant difference (*p* < 0.01) from immediate post-op to 24 h, and 72 h post-op (Table 1). The VAS values within the piezo and rotatory groups showed a statistically significant difference (*p* < 0.01) at postoperative 24 h and 72 h (Table 2). 

Inter-group comparison: CRP value in the piezo group was lower than in the rotary group, although the difference was not statistically significant (*p* > 0.05). The VAS value in the piezo group was significantly lower (*p* < 0.01) than in the rotatory group both post-op 24 h and 72 h (Table 3).

## 4. Discussion

The past decade has seen a surge in the number of innovative surgical tools and technology being introduced to decrease the surgical difficulty and postoperative morbidity associated with third molar surgery. An example of such an innovation is piezosurgery or the use of ultrasonic piezoelectric vibrations for relatively safer and precise osteotomies [20]. This method helps to perform precise osteotomy in areas even close to vital and important facial structures, including vessels and nerves [21]. The present study employed a split-mouth research design. The advantage of this research design is that it eliminates the patient’s compliance bias from the evaluated treatment effect, as elicited by Zhu et al. [22]. To standardize the results, the authors selected patients who had mandibular third molar impacted in a mesioangular class II position B (Pell and Gregory classification) [23]. The impaction selection made in the present study was based on the relatively higher frequency of the mesioangular class II position and the fact that there is published literature using a similar research design [20,24], which, in turn, would allow reliable comparisons. The statistics indicate that the value of CRP post-surgery is always more than the preoperative value, which is on par with Chander et al. [25], Desai et al. [26], and Hao Shen et al. [27]. This postoperative increase in the blood CRP (acute-phase protein) is a direct result of the local manipulation of the soft tissue and flap during the surgical extraction of impacted mandibular third molar procedures. 

Maria et al. [28] and Sanchis et al. [29] observed that the duration of the surgical procedure was directly related to postoperative tissue reactions and hence increased CRP. This is contrary to the present study results wherein the surgical technique with a longer time duration (piezo-average 50 min) showed lower CRP levels than the technique with a relatively short time duration (rotary-average 25 min). The conflicting data obtained could be explained by Ohzato et al. [30], who attributed the stimulation of inflammatory mediators to the degree of tissue destruction and the type of tissue. Thus, the higher CRP values obtained in the rotatory group could be a direct reflection of the amount of tissue destruction and the resulting influx of inflammatory mediators rather than surgical time. Calvo AM et al. and Larsen MK et al. also reported that CRP levels were not dependent on the duration of surgery. Smaller incisions, healing by primary intention, and the use of anti-inflammatory drugs were reported to be the major factors associated with low levels of CRP, while surgery-induced infection/aseptic traumatic inflammation was associated with increased CRP levels [31,32,33,34]. Bulut et al. [32] and El-Sharrawy [35] provided further evidence for the role of postoperative inflammation in increasing CRP levels, with the latter reporting a strong association between CRP levels and treatment response. Given the close association between CRP and postoperative morbidity, Lizuka and Lindqvist assessed the potential use of CRP as an early indicator of infection/aseptic inflammation. They reported that CRP was a relatively better indicator of inflammation than the erythrocyte sedimentation rate. Based on the abovementioned studies, CRP levels could be a vital tool for monitoring potential postoperative infection/aseptic inflammation following third molar surgery [32,35,36]. Also, a higher pre-operative CRP level could indicate a risk for prolonged severe postoperative inflammation. Thus, CRP recorded at the baseline (pretreatment) could be used to stratify the patient as high or low risk for potential postoperative morbidity. Prophylactic anti-inflammatory regimens could be prescribed according to CRP-based risk stratification [37].

The present study also evaluated postoperative pain. Published studies comparing post-surgical pain in piezosurgery and conventional rotatory surgery for mandibular third molar removal reported a relatively better outcome in the former [20,38,39,40,41]. Although piezoelectric surgery increased the treatment duration, it had a positive impact on the patients’ operative experience and postoperative clinical sequelae. The time taken for the piezosurgery group was more than for the rotatory group. The postoperative inflammation assessed clinically with swelling was less on the fifth postoperative day compared to the rotatory group. The trismus also showed a similar pattern in presentation in comparison. The time taken, however, was greater in the piezo group compared to the rotary group. There was a difference of 30 min more in the piezo group compared to the rotary group. This is on par with the present study, wherein postoperative pain (as recorded in the VAS) was higher in the rotary group than in the piezo group. This is the only landmark study that has worked on the quantitative analysis of C-reactive protein in patients undergoing two different surgical techniques to make a basis for scientific quantification. The inference of the study was that quantitative analysis of Crp was an excellent parameter for evaluating the use of piezosurgery in comparison to rotatory osteotomy. The CRP values, which were lower in Group A, were suggestive that it has a role in reducing inflammation when the surgery was done with piezosurgery. The ELISA technique of evaluation is a single quantitative method for grading the amount of inflammation when we are comparing two different methods of surgical removal of impacted third molars.

## 5. Conclusions

The results of the present study showed an increase in the blood CRP levels compared to the pre-operative values, although the rise dropped immediately at 24 h for the piezo group. In contrast, the CRP levels remained high even at 24 h in the rotary group. The rise in the C-reactive protein can be largely attributed to surgery-induced postoperative inflammation. The ability of piezo-based surgical interventions to spare the surrounding soft tissue could be the reason for the relatively lower CRP and VAS levels compared to the rotary group. Further multi-center studies with a larger sample size and longer follow-up duration are required to validate the findings of the present study.

## Figures and Tables

**Table 1:** Intragroup comparison of CRP across various time intervals using repeated-measures ANOVA.

Groups	Time	N	Mean	Standard Deviation	Standard Error	*p*-Value
Piezo CRP	Pre-op	25	3.71	4.594	0.919	0.976 #
	Immediate post-op	25	4.16	4.618	0.924	
	Post-op 24 h	25	3.97	4.754	0.951	
	Post-op 72 h	25	3.64	4.473	0.895	
	Total	100	3.87	4.545	0.455	
Rotatory CRP	Pre-op	25	2.83	1.774	0.355	0.000 **
	Immediate post-op	25	5.51	1.877	0.375	
	Post-op 24 h	25	12.60	3.793	0.759	
	Post-op 72 h	25	8.88	3.142	0.628	
	Total	100	7.45	4.588	0.459	

** statistically highly significant difference (*p* < 0.01); # non significant difference (*p* > 0.05).

**Table 2:** Intragroup comparison of VAS across various time intervals using the paired *t*-test.

Groups	VAS	Mean	N	Std. Deviation	Std. Error Mean	T Value	*p*-Value
Piezo	24 h	2.12	25	1.092	0.218	6.803	0.000 **
	72 h	0.32	25	0.476	0.095		
Rotary	24 h	3.92	25	1.256	0.251	14.025	0.000 **
	72 h	1.48	25	1.194	0.239		

** statistically highly significant difference (*p* < 0.01).

**Table 3:** Intergroup comparison of CRP and VAS between the groups using the *t*-test.

	Time Intervals	Groups	N	Mean	Standard Deviation	Standard Error	T Value	*p*-Value
CRP	Preop	Piezo	25	3.71	4.594	0.919	0.894	0.376 #
		Rotatory	25	2.83	1.774	0.355		
	Immediate post-op	Piezo	25	4.16	4.618	0.924	−1.350	0.183 #
		Rotatory	25	5.51	1.877	0.375		
	Post-op 24 h	Piezo	25	3.97	4.754	0.951	−7.096	0.000 **
		Rotatory	25	12.60	3.793	0.759		
	Post-op 72 h	Piezo	25	3.64	4.473	0.895	−4.794	0.000 **
		Rotatory	25	8.88	3.142	0.628		
VAS	Post-op 24 h	Piezo	25	2.12	1.092	0.218	−5.408	0.000 **
		Rotatory	25	3.92	1.256	0.251		
	Post-op 72 h	Piezo	25	0.32	0.476	0.095	−4.511	0.000 **
		Rotatory	25	1.48	1.194	0.239		

** statistically highly significant difference (*p* < 0.01); # non significant difference (*p* > 0.05).

## Data Availability

No data was used to support this study.
